# Stress and salivary cortisol levels among temporomandibular disorders: a case-control study

**DOI:** 10.22514/jofph.2025.039

**Published:** 2025-06-12

**Authors:** Lujain AlSahman, Hamad AlBagieh, Roba AlSahman

**Affiliations:** ^1^Oral Medicine and Diagnostic Sciences Department, College of Dentistry, King Saud University Riyadh, 57448 Riyadh, Saudi Arabia; ^2^Faculty of Dentistry, Royal College of Surgeons, D02YN77 Dublin, Ireland

**Keywords:** Cortisol, Facial pain, Inflammation mediators, Psychological stress, Saliva, Temporomandibular disorders

## Abstract

**Background**: This study investigated how cumulative lifetime stress, as measured by the Stress and Adversity Inventory (STRAIN) scale, relates to salivary cortisol levels in temporomandibular disorders (TMD) patients compared to controls. Furthermore, to determine which specific lifetime stress domains are the strongest predictors of TMD.** Methods**: The study was conducted with 110 participants (55 TMDs patients, 55 controls). Lifetime stress was assessed using the STRAIN questionnaire, and salivary cortisol levels were measured at two time points (7 AM and 10 AM) using Enzyme-Linked Immunosorbent Assay (ELISA). Statistical analyses included *t*-tests, Analysis of variance (ANOVA) and multiple regression to identify significant stress predictors for TMD.** Results**: The TMDs patients had significantly higher stress scores (11.10 ± 3.26) compared to the controls (1.43 ± 0.99) (*p* = 0.001). Myalgia showed highest stress levels (11.69 ± 3.72), while patients with myofascial pain had the lowest (8.80 ± 1.14) (*p* = 0.043). Cortisol levels were highest in the of disc displacement without reduction with limited mouth opening (DDWoR with LO) group (82.49 ± 124.34) and lowest in myalgia patients (4.69 ± 3.90) (*p* = 0.001). Significant stress predictors for TMDs included relationship stress (*p* = 0.04), humiliation (*p* = 0.02), marital/partner stress (*p* < 0.001) and death-related stress (*p* = 0.01).** Conclusions**: TMDs patients experience significantly higher lifetime stress and cortisol levels than controls. Myalgia patients showed a complex psychological and physiological stress link, whereas the DDWoR with LO subgroup exhibited a distinct physiological stress response. Specific life stressors, particularly relationship- and partner-related stress, are key predictors of TMDs. These findings reinforce the importance of a biopsychosocial approach in understanding and managing TMDs. Future research should focus on longitudinal and interventional studies to further elucidate causal mechanisms and effective therapeutic strategies.

## 1. Introduction

Temporomandibular disorders (TMDs) are a group of musculoskeletal conditions characterized by pain or discomfort in the preauricular region that affect the temporomandibular joint (TMJ) and its associated structures [1]. The symptoms of TMDs range from mild discomfort to severe myofascial pain, limited jaw movement [2, 3]. Recent research into TMDs has shifted from aetiology and treatment to a biopsychosocial model that integrates social, psychological and physical factors [4, 5]. Psychological factors such as anxiety and depression are increasingly recognized as important contributors to TMDs, particularly in individuals who experience chronic pain [6]. Thepsychological state of the patients and increased sensitivity to pain are two factors that are believed to play significant roles in the development and progression of painful TMDs [7].

Multiple studies have reported an association between stress and temporomandibular disorders (TMD) [8, 9, 10, 11]. A central mechanism underlying this relationship involves the hypothalamic-pituitary-adrenal (HPA) axis. Stress activation triggers the HPA axis, resulting in the secretion of cortisol, the primary stress hormone [12]. While the acute release of cortisol is an adaptive response that aids in coping with stress, chronic stress can lead to dysregulation of the HPA axis [13].

Although stress is strongly linked to the TMD, the precise mechanisms through which this occurs remain unclear [14, 15, 16, 17]. The use of physiological markers to evaluate conditions that could be related to psychosocial stress has grown over time [14]. Cortisol, as a physiological marker of stress, has been extensively studied in various stress-related conditions [18]. However, there is lacking appropriate evidence on the role of cortisol and stress in the TMD pathophysiology [19]. Previous studies have underscored the necessity of exploring how psychological stress manifests physiologically in individuals with TMD [16, 19]. Additionally, there is a growing interest in examining specific stress characteristics and domains to better understand their impact on TMD [4, 15, 20].

The Diagnostic Criteria for Temporomandibular Disorders (DC/TMDs) is the primary standardized tool for the diagnosis of TMDs; it incorporates axis I for physical evaluation and axis II for psychological assessments [21]. While axis II includes psychosocial assessments, the focus is on symptoms that have occurred within the past two weeks, and it does not account for lifetime stress, including early life stressors. Although various psychological scales have been used in TMDs research, previous research recommends the use of the Stress and Adversity Inventory (STRAIN) scale to assess stress in clinical populations, including TMDs [4]. The STRAIN scale provides an assessment of lifetime stress across primary life domains that impact overall health and quality of life [22]. It includes both acute life events and chronic difficulties associated with various health implications.

Cortisol is a glucocorticoid hormone that regulates metabolism, immune response and stress adaptation [12]. It functions as both an anti-inflammatory and pro-inflammatory agent, depending on the body’s physiological state [23, 24]. Under chronic stress, the influence of cortisol veers towards pro-inflammatory effects; thus, it contributes to dysregulation in pain and stress pathways [23]. Given its role as a biomarker of chronic stress, the measurement of salivary cortisol provides a non-invasive method to assess the body’s cumulative stress. The null hypothesis is that there is no significant difference in cortisol and stress levels in TMDs patients.

This study objective is to investigate how cumulative lifetime stress, as measured by the STRAIN questionnaire, relates to the salivary cortisol levels in TMDs patients compared to controls.

In addition, the study seeks to determine which specific lifetime stress domains are the strongest predictors of TMD.

To address these objectives, the study focused on the following research question:

How does cumulative lifetime stress, as measured by the STRAIN questionnaire, relate to salivary cortisol levels in individuals with TMDs compared to healthy controls?

## 2. Materials and methods

### 2.1 Study design

This case-control study was conducted as part of a multidisciplinary investigation of TMDs patients within the framework of a PhD project at the Dental University Hospital, King Saud University, Riyadh, Saudi Arabia. The study protocol was approved by the Institutional Review Board (IRB) at King Saud University under project number E-22-7168, issued on 11 September 2022, in accordance with the ethical principles outlined in the Declaration of Helsinki. The study followed the STROBE guidelines [25]. Written informed consent was obtained from all participants. This study applied the PECO framework, with TMDs patients and healthy controls (population), lifetime stress (exposure) and salivary cortisol levels (outcome), to examine the relationship between stress and cortisol in both groups.

### 2.2 Selection criteria

A total of 110 participants were prospectively recruited between November 2022 and April 2023. The study included a TMDs group (n = 55) and a gender- and age-matched control group (n = 55). All participants were informed regarding the study, and written consent was obtained.

The inclusion criteria required participants to be adults aged between 18–40 years. The TMDs group consisted of individuals diagnosed with symptomatic disc displacements (DDs) and/or myalgia according to the Diagnostic Criteria for Temporomandibular Disorders (DC/TMD). The control group comprised individuals visiting the Dental University Hospital for routine dental check-ups. Participants were excluded if they (1) had conditions that affect pain sensitivity (*e.g.*, fibromyalgia, rheumatoid arthritis, autoimmune diseases, migraines, neurological or neuropsychiatric disorders); (2) had used anti-inflammatory drugs, opioids, analgesics or steroids within the past 30 days (unless they had discontinued use); (3) were taking medications that affect saliva secretion (*e.g.*, calcium channel blockers, antidepressants, antihistamines); (4) were pregnant or lactating, obese, or smokers; (5) had salivary gland diseases (*e.g.*, tumours, stones, hyposalivation); (6) had complaints of dry mouth, edentulism or prosthodontic rehabilitation (complete/partial dentures); (7) had poor oral hygiene (Plaque or Gingival Index >2.0) or severe periodontal disease (clinical attachment loss ≥5 mm, probing depth ≥6 mm, significant bone loss); (8) had untreated or actively treated mental health disorders (*e.g.*, depression, anxiety, post-traumatic stress disorder (PTSD); (9) had obstructive sleep apnoea (OSA); (10) had malignancies with ongoing radiotherapy/chemotherapy; or (11) had adrenal hyperfunction or Cushing’s disease. Participants were from the same cohort that was used in a prior study [26].

### 2.3 Clinical evaluation

The Diagnostic Criteria for Temporomandibular Disorders (DC/TMD) tool was published in 2014. The tool establishes standardized and valid diagnostic criteria for clinical and research settings and provides a globally recognized framework to be followed for both clinical and research purposes [21].

A thorough assessment of the participants’ jaw function and related structures was conducted. The clinician measured maximum unassisted and assisted mouth opening, along with lateral and forward movements, to evaluate range of motion and detect any restrictions. While the patient performed these movements, the temporomandibular joints (TMJs) were examined for clicking, popping or crepitus sounds. The masticatory muscles were palpated to check for tenderness or referred pain. The TMJs were also palpated, both at rest and during movement.

### 2.4 STRAIN questionnaire

The Stress and Adversity Inventory for Adults (STRAIN) questionnaire was used to assess stressors throughout the lifespan. This is a validated, structured interview-based scale designed to assess an individual’s lifetime stressors across multiple life domains (https://is.gd/i3VlNT). The questionnaire categorizes the stressors into the following domains: housing, financial, work, relationship, humiliation, marital/partner, health/treatment, death, physical danger. This scale has demonstrated excellent test-retest reliability, discriminant validity, and good concurrent and predictive utility for several stress-related health outcomes, including anxiety and depression [22]. As per the recommendations of the Institutional Review Board (IRB) and in consideration of cultural sensitivities, two questions, (19) “Were you assaulted or attacked (*e.g.*, someone tried to hurt, molest, or rape you)” and (20) “Have you experienced ongoing sexual abuse (*e.g.*, rape, molestation or unwanted sexual contact)”, were removed from the questionnaire. Any discussion of such topics is highly sensitive in this study population, where participants may be unwilling to disclose such experiences. This exclusion was specifically requested by the IRB to protect participant’s well-being and respect their autonomy and comfort.

The interviews were conducted in a controlled clinical setting at the Dental University Hospital. Each interview was conducted in a quiet and private consultation clinic, with only the researcher and the participant present. The seating arrangement provided adequate space to ensure a relaxed setting. Before the interview commenced, the participants received a detailed explanation of the study’s purpose and procedures. Each session lasted approximately 20 minutes, and participants were provided with time to reflect on their responses, if necessary.

### 2.5 Saliva collection

The saliva sample collection process was meticulously organized to ensure accuracy and consistency from the clinic to the laboratory. The participants were asked to not brush their teeth, eat or perform activities that could cause blood contamination before collection. After one minute of rest, participants were requested to rinse their mouths thoroughly with water to remove any debris. Two millilitres of unstimulated saliva were collected in pre-graduated polypropylene vials with conical-bottom centrifuge tubes and immediately stored at −20 °C in ultra-low temperature freezers (TSX60086A, ThermoFisher Scientific, Waltham, MA, USA). Upon arrival at the laboratory, the saliva samples were kept at room temperature for 10 minutes. The samples were centrifuged in an Andreas Hettich GmbH & Co. KG centrifuge (EBA 270, Tuttlingen, BW, Germany) at 3000 Revolutions Per Minute (RPM) for 5 minutes. For cortisol conjugation, the salivary samples were reconstituted using 16 μL of sterile dH_2_O.

Salivary cortisol levels were analysed for all samples using an ELISA kit (RE52071, IBL International Corporation, Lucerne, LU, Switzerland) under consistent conditions for both the control and TMDs groups [26]. All collected information was systematically entered into SPSS Version 23.0 (IBM, Armonk, NY, USA).

### 2.6 Statistical analysis

A power analysis (α = 0.05, β = 0.80) determined a minimum required sample size of 50 per group for the detection of medium effect sizes. Descriptive statistics mean ± Standard deviation (SD) were used for demographic and clinical data. Normality was confirmed using the Kolmogorov-Smirnov test.

The demographic and clinical data obtained from the participants were recorded and transferred into the digital database (Microsoft Excel 2020). Frequencies, mean values and standard deviations (SDs) were obtained for the included variables. Comparisons between groups were conducted using the Chi-square test. An independent *t*-test was utilized to calculate the mean STRAIN score.

The Z-score transformation was applied for cross-comparisons between stress and cortisol levels. The results were considered significant if the *p*-value was ≤ 0.05. The data were analysed using IBM SPSS Statistics (ver. 23.0 for Windows; IBM Corporation, Armonk, NY, USA). One-way ANOVA with Tukey’s *post hoc* test was employed to compare stress scores among different TMDs subtypes. Cronbach’s alpha was used to assess the reliability of the scale by determining how well its items correlate with one another. A higher alpha value indicates strong internal consistency.

To account for multiple comparisons in the multivariable regression analysis, the Bonferroni correction was applied, with the adjusted significance threshold set at α = 0.05/10 = 0.005. *p*-values were adjusted accordingly, and only variables with adjusted *p*-values below this threshold were considered statistically significant.

## 3. Results

The Stress and Adversity Inventory for Adults (STRAIN) scale demonstrated high internal consistency, with a Cronbach’s alpha value of 0.933. This indicates that the scale effectively captured lifetime stress in this study, ensuring the reliability and validity of the collected stress-related data.

### 3.1 Sociodemographic characteristics

This study included 110 participants, all of whom underwent clinical examination for TMDs according to the Diagnostic Criteria for Temporomandibular Disorders (DC/TMD). The participants were aged between 18–40 years, with a male-to-female ratio of 1:2. In the study group, the majority of the participants were aged between 18–30 years (53.1%), whereas the majority of the control group were aged between 31–40 years (52.5%). The majority in both groups had college degrees (study: 47.5%, control: 52.5%) and were married (study: 44.4%, control: 55.6%).

### 3.2 Stress scale

A comparison of STRAIN scores between the groups demonstrated a statistically significant difference (*p* = 0.001), with the study group exhibiting higher stress levels than the controls (11.1091 ± 3.26). The mean score was higher among males (6.37 ± 6.10; *p* = 0.91) than females. Significant variations in stress levels were observed among the subgroups.

The myalgia group had the highest mean STRAIN score (11.69 ± 3.72), while the myofascial pain group had the lowest mean score (8.80 ± 1.14). The disc displacement without reduction with limited mouth opening (DDWoR with LO) group was positioned in between, with a mean score of (11.10 ± 1.52), which reflected a distinct stress pattern (*p* = 0.043).

Higher mean scores were noted among participants aged between 31–40 years (6.52 ± 5.46), those with college degrees (6.51 ± 5.43) and those who were married (6.59 ± 5.27). However, none of the demographic variables (gender, age, education or marital status) showed statistically significant differences in STRAIN scores (*p* > 0.05) (Table 1). Stressors, including acute life events and chronic difficulties, were significantly associated with TMDs (*p* < 0.001). Specific stressors related to housing, treatment/health, marital/partner relationships, humiliation and other interpersonal relationships were significantly associated with TMDs (*p* < 0.001).

**Table 1.** Comparing the STRAIN scores based on demographics and groups.

Category	Group	N	Mean STRAIN Score	SD	*p*-value
Gender					
	Male (M)	30	6.37	6.10	0.91
	Female (F)	80	6.24	5.18	
Age (yr)					
	18–30	49	5.96	5.40	0.59
	31–40	61	6.52	5.46	
Education					
	High school	9	3.56	4.82	0.12
	College degree	101	6.51	5.43	
Marital Status					
	Married	90	6.59	5.27	0.20
	Single	20	4.85	5.98	
Group					
	Control	55	1.43	0.99	0.001*
	Study	55	11.10	3.26	
Subgroups					
	Myalgia	35	11.69	3.72	0.043*
	Disc displacement without reduction (LMO)	10	11.10	1.52	
	Myofascial pain	10	8.80	1.14	

*Statistical significance. STRAIN: Stress and Adversity Inventory; SD: Standard Deviation.

### 3.3 Salivary cortisol levels and TMDs subgroups

Table 2 (Ref. [26]) presents the mean salivary cortisol levels of the TMDs subgroups and the control group. These values represent the average of saliva samples collected at both intervals (early and late morning). Disc displacement without reduction with limited mouth opening showed the highest cortisol levels, with a value of 82.49 ± 124.34 (8.32–398.64). Patients with myofascial pain had a mean cortisol level of 13.03 ± 8.46 (5.69–34.68), while those with myalgia had a mean of 4.69 ± 3.90 (0–15.53) [26].

**Table 2.** Comparison of cortisol levels among TMDs subgroups and control group [26].

Group	Mean	SD	Minimum	Maximum	*p* value
Disc displacement without reduction with limited mouth opening	82.49	124.34	8.32	398.64	0.001*
Myofascial pain	13.03	8.46	5.69	34.68	
Myalgia	4.69	3.90	0.00	15.53	
Control	8.94	13.80	0.00	92.90	

SD: standard deviation. *Statistical significance.

### 3.4 Stressor domains

Before applying multiple comparison correction, six predictors were statistically significant (*p* < 0.05). However, after applying a Bonferroni correction, only four predictors remained significant: relationship stress (adjusted *p* = 0.04), humiliation (adjusted *p* = 0.02), marital/partner stress (adjusted *p* < 0.001) and death-related stress (adjusted *p* = 0.01). Housing and health/treatment stress were initially significant (*p* = 0.005 and *p* = 0.039, respectively) but did not meet the adjusted significance threshold (0.005).

The regression analysis, with TMDs as a dependent variable, showed several significant predictors. Significant domains included housing (B = −1.644, *p* = 0.005), relationship (B = −1.372, *p* = 0.004), humiliation (B = 1.860, *p* = 0.002), marital/partner, (B = 2.337, *p* < 0.001), health/treatment (B = 1.736, *p* = 0.039) and death (B = −1.582, *p* = 0.001). These findings indicate that the humiliation and partner domains had the strongest positive correlations with TMD. Other variables, including financial, work, crime and physical danger domains, were not significant predictors (*p* ≥ 0.05). Overall, the model explained 61.8% of the variance in the TMD, with an adjusted *R*^2^ of 42.7% (*p* = 0.001) (Table 3).

**Table 3.** Multivariable analysis with the TMDs subgroup as dependent variables and all the lifetime stressors as independent variables.

Predictor Variable	Unstandardized Coefficients (B)	Std. Error	Standardized Coefficients (Beta)	*t*-Value	Sig. (*p*-value)	Adjusted *p*-value	95% CI (Lower)	95% CI (Upper)
Housing	−1.644	0.549	−0.410	−2.996	0.005	0.05 (NS)	−2.756	−0.532
Financial	−0.025	0.490	−0.009	−0.052	0.959	1.00	−1.016	0.966
Work	0.554	0.471	0.201	1.175	0.248	1.00	−0.400	1.508
Relationship	−1.372	0.446	−0.549	−3.075	0.004	0.04*	−2.273	−0.471
Humiliation	1.860	0.554	0.525	3.359	0.002	0.02*	0.737	2.983
Partner	2.337	0.562	0.935	4.159	0.001	0.01*	1.198	3.476
Crime	−0.779	0.462	−0.310	−1.684	0.101	1.00	−1.716	0.158
Health	1.736	0.812	0.315	2.138	0.039	0.39 (NS)	0.087	3.385
Death	−1.582	0.447	−0.468	−3.536	0.001	0.01*	−2.486	−0.678
Physical danger	0.584	0.719	0.106	0.812	0.422	1.00	−0.873	2.041

NS: No longer significant after correction; Std. Error: Standard Error; Sig: Significant; CI: Confidence Interval. *Statistical significance.

## 4. Discussion

This study examined the relationship between lifetime stress and salivary cortisol levels in individuals with TMDs and healthy controls. The focus was on variations among TMDs subgroups and stress patterns. The null hypothesis cannot be accepted, as a significant difference in stress and cortisol levels was found between the TMDs and control groups. The findings indicate that individuals with TMDs had significantly higher lifetime stress and cortisol levels compared to the controls. This aligns with the findings of previous research showing high cortisol levels among TMDs patients.

High stress levels in TMDs groups are supported by existing research, which has reported a strong association between stress and TMDs [4, 27, 28]. Moreover, the results show significant variability in the stress and salivary cortisol levels among TMDs subgroups.

The myalgia group exhibited elevated stress and low cortisol levels, which contradicting with previous findings indicating higher cortisol concentrations in muscle-related TMDs [29]. While TMDs were indeed associated with psychological problems in all cases, those with muscular conditions appear to be the most psychologically affected subgroup [30]. One proposed mechanism for TMDs muscle-related conditions is masticatory muscle hyperactivity, where stress induces heightened activation of the muscles, potentially leading to parafunctional habits or centrally mediated responses [31]. The myofascial pain group showed moderate elevations in both stress and cortisol levels. Previous studies have reported that, compared to muscular conditions, disc displacements conditions are less directly influenced by psychological stress [32].

In this study, a disassociation between stress and cortisol levels was observed in the group of disc displacement without reduction with limited mouth opening (DDWoR with LO). The findings indicate that participants with DDWoR with LO demonstrate moderate stress levels despite elevated cortisol. Some authors believe that, in this subgroup, cortisol may function as an inflammatory marker associated with DDWoR with LO pathophysiology rather than a direct indicator of psychological stress. These results highlight the importance of interpreting cortisol within the broader context of TMDs, as it is a key biomarker [33] that can indicate both psychological and inflammatory markers, which vary across TMDs subgroups. The inclusion of a stress scale is beneficial for the comparison of cortisol and for gaining deeper insight into cortisol’s role in the pathophysiology of each subgroup.

Future research should further investigate the relationship between inflammatory processes and cortisol dynamics in TMDs subgroups to enhance the understanding of the role of cortisol in the disease progression.

Another explanation could be chronic pain-induced dysregulation of the hypothalamic–pituitary–adrenal (HPA) axis [14]. Long-term restricted mandibular movement in the LMO subgroup may cause sustained nociceptive activation, which may in turn result in persistent cortisol release.

It has been demonstrated that chronic pain interferes with the HPA axis, which then maintains high cortisol levels even in the absence of subjective stress perception. Chronic pain can lead to persistent physiological arousal, independent of emotional distress [34]. Furthermore, pain may act as a physiological stressor, activating the autonomic nervous system, which leads to elevated cortisol levels. Functional limitations associated with LMO may impose a continuous physiological burden, which will drive stress-related cortisol secretion irrespective of emotional distress. This suggests that while stress may not be the primary driver of disc displacement (DD), pain-related physiological stress responses could modulate cortisol levels in this subgroup [35]. In addition, patients with DDWoR with LO may develop adaptive coping mechanisms that reduce subjective stress perception while maintaining persistent physiological stress responses, which explains why cortisol levels remain elevated despite moderate reported stress. This suggests a disconnect between psychological perception and physiological markers [36]. Moreover, central sensitization and neuroinflammation processes may contribute to sustained cortisol activation in chronic pain. Heightened nervous system excitability in TMDs patients could exaggerate physiological stress responses, even in the absence of perceived psychological distress [13].

Lifetime stressors domains were significant predictors in TMDs participants compared to controls. These findings reinforce the biopsychosocial nature of TMDs and their sensitivity to various life stressors [6, 15, 37]. Speculand *et al*. [38] reported that TMDs patients experienced nearly twice as many stressful life events as controls, with work, financial and health-related challenges frequently contributing to the onset of TMDs. People who experience stressful life events are more susceptible to the development and progression of TMDs [39, 40]. Stressors involving family or friends significantly impacted on TMD, which is consistent with research that has shown that traumatic experiences, such as injuries or losses involving loved ones, are significant in TMDs patients [41]. Unlike some studies [20, 42], the financial-related stressors were not significant predictors of TMDs in this study population. This is possibly due to cultural or demographic factors. In contrast to these findings, financial stressors and TMDs have shown a strong correlation in other populations, with low income being linked to the presence of TMDs [42].

### 4.1 Limitation

Sample size limitations pose a challenge, particularly in the TMDs subgroups, which may limit the generalizability of the findings, which should be interpreted with caution. The exclusion of two questions may limit cross-cultural comparability. Since cortisol fluctuates throughout the day, the inclusion of additional afternoon and evening measurements in future studies could provide more comprehensive insight.

### 4.2 Recommendations for future studies

Future studies with larger cohorts are needed to validate these results.

These studies should collect cortisol samples at multiple time points (morning, afternoon, evening) for more robust cortisol analysis. The sample should be expanded to include different age groups and socioeconomic backgrounds. Different scales that capture a wide range of stressors could be used in future studies.

## 5. Conclusions

Individuals with TMDs had significantly higher lifetime stress compared to controls. Myalgia patients showed the highest stress and low salivary cortisol levels, which indicates complex connection between psychological and physiological factors. The elevated cortisol levels in the DDWoR with LO are more indicative of an inflammatory marker rather than a stress response, as stress levels remain low. Salivary cortisol is an important biomarker in TMDs, reflecting both psychological status and inflammatory activity, depending on the TMDs subgroup. The authors believe that cortisol dual role provides insight into the pathophysiology of TMDs.
